# An Impactful Summer

**DOI:** 10.1016/j.identj.2022.08.005

**Published:** 2022-08-25

**Authors:** Lakshman Samaranayake

**Affiliations:** Editor-in-Chief

A number of important events related to your journal transpired over the summer months. Of these, the most seminal event could be regarded as the publication of the *International Dental Journal (IDJ)* supplement on “Oral Health for an Ageing Population.”^1^ This was a landmark compendium highlighting the varied aspects of the effect of ageing on the maxillofacial complex. Written by an internationally renowned group of erudite authors, the articles^2^, ^3^, ^4^, ^5^, ^6^ describe the burden of oral diseases and access to oral care in an ageing society as well as the decline in oral function with ageing and how these could be mitigated. Furthermore, this unique compilation tackles the thorny question of policy changes required globally to address a glaring health issue^5^ that we all will inevitably face within the next few decades.

It is no secret that, globally, the older population has been growing exponentially, with the prospect that by 2050 a quarter of the world's population—around 2 billion—will be older than 60 years. Unfortunately, older persons can be particularly affected by poor oral health and may be more susceptible to diseases such as caries, periodontitis, dry mouth, and tooth loss, thus impacting their general health and overall quality of life. I believe that the *IDJ* supplement will be an outstanding contemporaneous contribution to dentistry, and it is essential reading for dental practitioners, academics, and policymakers—in short, all stakeholders in dentistry.

Another important feature we initiated over the summer months was the introduction of a new article type entitled “Succinct Rapid Communications” (SRC) that will supplement the extant spectrum of *IDJ* articles. These are intended to be reports of fully developed primary research, yet considerably shorter than a standard article published in other sections of the journal. The SRC pathway is particularly suitable for postgraduate researchers with rapid publication deadlines necessitated by time-constrained, graduation requirements. In addition, as the name implies, we shall consider rapid publication of these articles, and there will be a considerably lower article processing charge than that of the standard *IDJ* articles.

Last but not least, this summer has also witnessed the reemergence of other viral diseases, on top of the lingering COVID-19 pandemic. These include the resurfacing of monkeypox in the West, an old disease prevalent mainly in the African subcontinent, in addition to perhaps polio being noted in the Americas. Monkeypox, though not as contagious as COVID-19, may have implications for dentistry, and the current *IDJ* issue contains a comprehensive article on monkeypox and its implications for dentistry.^7^ In addition, along the same theme, we bring to the reader 5 more articles on varied aspects of COVID-19 and dentistry. These range from oral mycoses seen in COVID-19, to the indirect, collateral damage due to the COVID -19 pandemic consequential to delayed visits to the dentist and the resultant neglect of other oral diseases, such as cancers.

We trust you will enjoy perusing the current issue of *IDJ* and, of course, the freely available aforementioned supplement on healthy ageing,^1^, ^2^, ^3^, ^4^, ^5^, ^6^ which is a brilliant article collection!
